# Unveiling Bifunctional Hydrogen Bonding with the Help of Quantum Chemistry: The Imidazole-Water Adduct as Test Case

**DOI:** 10.1021/acs.jpca.1c01679

**Published:** 2021-04-05

**Authors:** Alessio Melli, Vincenzo Barone, Cristina Puzzarini

**Affiliations:** † Scuola Normale Superiore, Piazza dei Cavalieri 7, 56126 Pisa, Italy; ‡ Dipartimento di Chimica “Giacomo Ciamician”, Università di Bologna, Via Selmi 2, 40126 Bologna, Italy

## Abstract

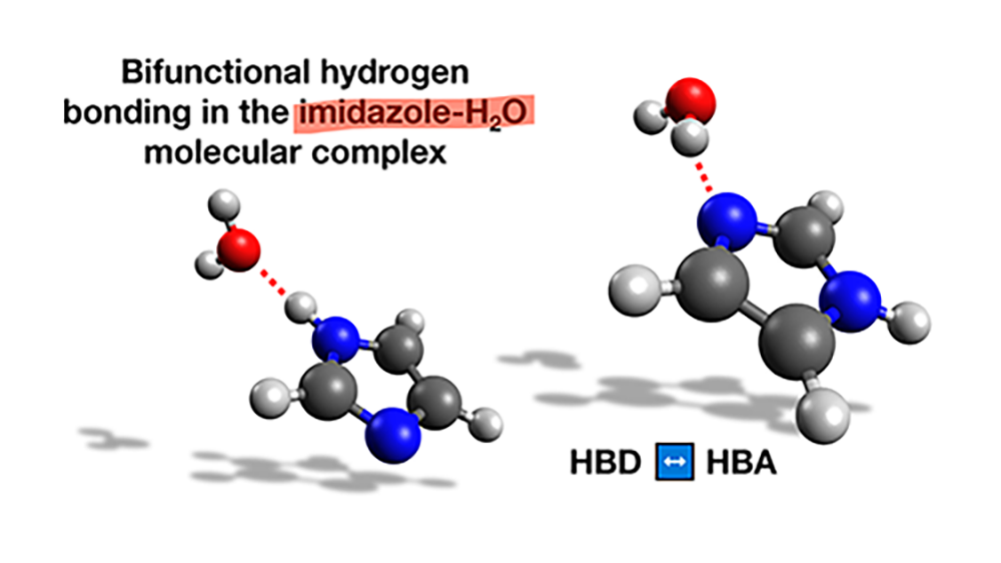

The ubiquitous role of water and its amphiprotic nature call for a deeper insight into the physical–chemical properties of hydrogen-bonded complexes formed with building blocks of biomolecules. In this work, the semiexperimental (SE) approach combined with the template model (TM) protocol allowed the accurate determination of the equilibrium structure of two isomeric forms of the imidazole-water complex. In this procedure, the integration of experiment (thanks to a recent rotational spectroscopy investigation) and theory is exploited, also providing the means of assessing the reliability and accuracy of different quantum-chemical approaches. Overall, this study demonstrated the robustness of the combined SE-TM approach, which can provide accurate results using affordable quantum-chemical methods. Finally, the structural and energetic characteristics of these complexes have been examined in detail and compared with those of analogous heterocycle–water adducts, also exploiting energy decomposition analyses.

## Introduction

In recent years, the number of joint experimental–computational studies on noncovalent complexes has significantly increased, with the aim of obtaining a deeper knowledge of the underlying interactions.^1−9^ The interest is often focused on spectroscopic and structural properties as well as on energetic characterizations, the latter often being coupled with their quantitative interpretation in terms of chemically meaningful concepts (e.g., electrostatics, induction, dispersion, etc.).^2,3,6,7,9^

Among different experimental techniques, rotational spectroscopy is the most suitable to derive structural information owing to the direct connection between rotational constants and molecular structure.^10^ Rotational spectroscopy is indeed a powerful tool for molecular structure determinations, which are the mandatory prerequisites for investigating structure–activity relationships as well as deriving chemical and physical properties.^11−15^ Furthermore, rotational spectroscopy investigations are performed in the gas phase, thus allowing one to focus on intrinsic effects without the perturbations introduced by the environmental effects operative in condensed phases.

Accurate structural determinations can be performed by exploiting the semiexperimental (SE) approach, which relies on extracting from the experimental outcomes the equilibrium structure details using quantum-chemical (QC) computations for providing the missing information.^16^ Going more in detail, SE equilibrium structures can be obtained from a least-squares fit (LSF) of the SE equilibrium rotational constants, in turn derived from the experimental ground-state counterparts by subtracting the computed vibrational corrections.^16^ Whenever experimental information is available for a sufficient number of isotopologues, a complete structural determination is possible. For small- and medium-sized organic and biological molecules, several studies have shown that vibrational corrections computed using hybrid or, even better, double-hybrid density functionals in conjunction with suitable basis sets have the required accuracy to obtain reliable results.^17,18^ The application of such an approach has led to the development of a database of SE equilibrium structures containing information for an increasing number of species, and which already incorporates the most relevant prebiological building blocks.^17,18^

Moving to molecular complexes, it becomes particularly challenging to obtain a set of experimental data sufficient for a complete structural determination. In particular, the most difficult information to retrieve is the position of hydrogen atoms because a full set of deuterated species is rarely experimentally available. In all cases where there are missing data, the usual approximation is to fix the intramolecular parameters at those of the isolated fragments and to fit only a limited number of intermolecular parameters. Unfortunately, this approximation is not free from difficulties, especially when flexible fragments are involved, and more advanced approaches are needed. To overcome this issue, we propose an extension of the template model approach (TMA),^17^ which consists of correcting the structural parameters of a complex system, obtained at a suitable QC level, by using the corresponding SE values of suitable fragments, referred to as template models (TMs).^19^ In the case of noncovalent molecular complexes, the TMs are the monomers, whose SE equilibrium structures are likely available in the above-mentioned database. The intramolecular parameters are greatly improved by the exploitation of the TMA, and they can thus be confidently kept fixed. Then, the number of experimental data becomes sufficient to optimize the intermolecular parameters.

Whenever some SE equilibrium intermolecular parameters cannot be determined, they can be obtained from partial geometry optimizations at an accurate QC level. On the basis of recent works,^20−22^ the so-called jun-“cheap” composite scheme (hereafter jun-ChS) provides, for noncovalent complexes, interaction energies on par with the most sophisticated composite methods at a significantly reduced computational cost. It is, therefore, natural to investigate if this computational approach is able to also deliver accurate geometrical parameters to be employed in the interpretation and prediction of equilibrium rotational constants. In the perspective of studying even larger systems, the double-hybrid DSD-PBEP86 functional (in the recent reparametrization by Martin and co-workers,^23^ rev-DSD-PBEP86) in conjunction with the jun-cc-pVTZ basis set^24,25^ appears particularly promising and will be compared to jun-ChS results.

Once the methodology is defined and the computational level is selected, the next step is the choice of a challenging, yet representative, test case to assess the methodology. Among different noncovalent interactions, hydrogen bonds play a central role, especially for biological systems. Therefore, the interaction between building blocks of biomolecules and water represents a natural starting point for our analysis, also in view of the ubiquitous presence and the amphiprotic character of water. Focusing on the potential partner of the molecular complex, heterocyclic compounds appear interesting in view of the prebiotic character of the species containing the C–N moiety as well as their presence in important biological molecules. In this framework, imidazole is particularly appealing because of the concomitant presence of donor and acceptor sites.

**Figure 1:**
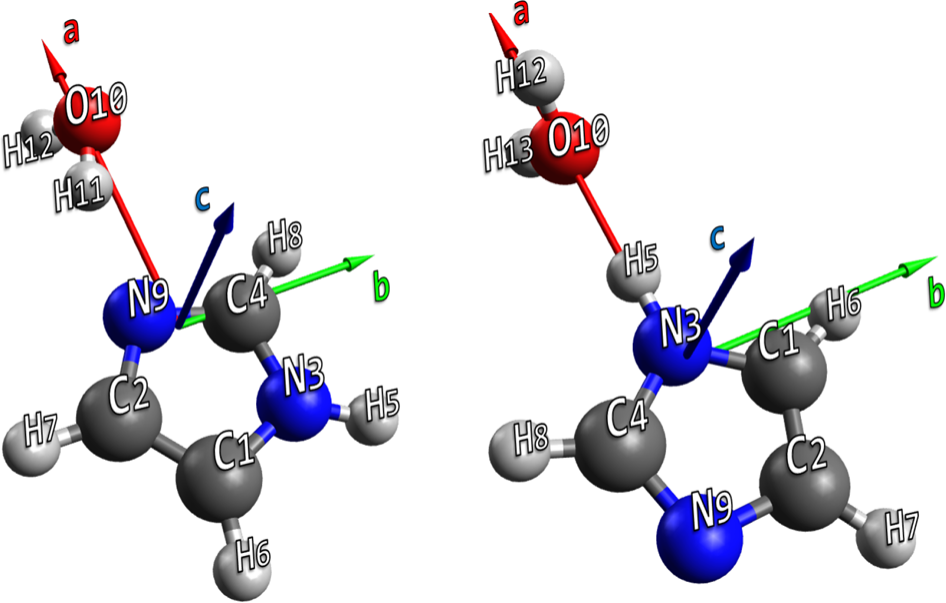
H_2_O–Imid (left) and Imid–H_2_O (right) structures (rev-DSD level) together with the atom numbering.

In conclusion, this study will address the characterization (by means of the methodologies mentioned above) of the water–imidazole complex, whose rotational spectrum has recently been reported for a number of different isotopologues.^26^ Interestingly, two different adducts have been experimentally observed (see Figure 1) in which water plays the role of either hydrogen bond (HB) donor (HBD) or acceptor (HBA).^26^ On the contrary, in a previous experimental investigation with supersonic jet FTIR spectroscopy, only the HBD complex was detected, thus suggesting that it is significantly more stable than the HBA counterpart.^27^ However, the study of ref (26) was not able to experimentally determine the relative stability of the two adducts. For this reason, an accurate energetic characterization is also deserved.

This manuscript is organized as follows. In the next section, the computational methodology is presented in all details. The following section will report and discuss the results of structural and energetic investigations. Finally, concluding remarks will be provided.

## Computational Methodology

The equilibrium structure of both isomers of the imidazole–water molecular complex (see Figure 1) has been evaluated by exploiting QC composite schemes as well as the SE approach. Both of them are described with some details in the following.

As briefly mentioned in the Introduction, within the SE methodology, the equilibrium structural parameters of the investigated system are determined from a LSF of the equilibrium moments of inertia of different isotopologues.^16^ However, laboratory measurements give access to vibrational ground-state rotational constants (*B*_0_^*i*^, where *i* refers to the inertial axes *a*, *b*, *c*), thus requiring the vibrational corrections (*ΔB*_vib_^*i*^) to be subtracted in order to obtain the corresponding SE equilibrium rotational constants (*B*_e_^*i*^).^16^ From the latter, the SE equilibrium moments of inertia are straightforwardly derived, these being inversely proportional to rotational constants.^10^

To better clarify the statements above, it has to be noted that, in the framework of vibrational perturbation theory to the second order (VPT2),^28^ the equilibrium rotational constants can be expressed as
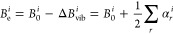
1where the α_*r*_’s are the vibration–rotation interaction constants and the sum runs over all *r* vibrational modes. Noted is that the evaluation of the α_*r*_’s implies anharmonic force field calculations (for details, see, e.g., refs (29−32)).

To recap, in the SE procedure, the second term on the right-hand side of eq 1 (i.e., *ΔB*_vib_^*i*^) is derived from QC calculations, while the first term is obtained from the experiment. *ΔB*_vib_^*i*^ being significantly smaller than *B*_*e*_^*i*^, the former term can be determined at an affordable level of theory (such as methods rooted in the density functional theory, DFT) without significantly affecting the accuracy of the resulting SE equilibrium rotational constants.^11,18,29,33^ The calculation of the α_*r*_’s from DFT anharmonic force fields led to the collection of accurate SE equilibrium geometries for several systems, ranging from isolated molecules to clusters.^3,6,9,17,18,34−38^ In this work, the global-hybrid B3LYP functional,^39,40^ also incorporating the Grimme’s DFT-D3 scheme^41^ for the treatment of dispersion effects in conjunction with the Becke–Johnson (BJ) damping function,^42^ has been used for the calculation of the vibrational contributions. The partially augmented double-ζ jun-cc-pVDZ basis set^24,25^ has been employed in combination with B3LYP-D3(BJ). Hereafter, this level of theory is shortly referred to as B3.

The starting point of the LSF procedure is a guess geometry obtained by means of the TMA.^18^ In the present context, the TM species are the monomers, imidazole and water, and their structural parameters within the complex have been adjusted using the SE equilibrium geometry of the isolated fragments.^a^ For a generic intramolecular equilibrium structural parameter *r*, which has been optimized at the *x* level of theory, the TMA parameter (*r*_TMA_) is calculated as
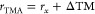
2where
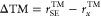
3In eqs 2 and 3, TM is the isolated fragment. The rev-DSD-PBEP86-D3(BJ) functional^23^ in conjunction with the jun-cc-pVTZ basis set^24,25^ (hereafter denoted as rev-DSD) has been considered as the *x* level of theory in the equations above because it is proven to offer a very good description of noncovalent complexes.^43^

In addition to the SE approach, the equilibrium structure of the molecular complex has been evaluated using the so-called “cheap” composite scheme (hereafter ChS).^44^ The original version of this accurate yet cost-effective approach for medium-sized systems^45,46^ is the following:^44^
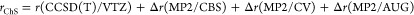
4The terms on the right-hand side are presented here below in order of appearance:

i. The starting point is the coupled-cluster ansatz including single and double excitations with a perturbative treatment of triples, CCSD(T),^47^ within the frozen-core (fc-) approximation and in conjunction with the cc-pVTZ basis set.^24^
ii. The correction due to the extrapolation to the complete basis set (CBS) limit is evaluated using the *n*^–3^ formula^48^ and applied to the geometrical parameters optimized using Møller–Plesset perturbation theory to second order^49^ (fc-MP2). The cc-pV*n*Z (with *n* = T,Q) basis sets are employed.
iii. The core–valence (CV) correlation contribution is calculated as the difference between all-MP2/cc-pCVTZ^50^ and fc-MP2/cc-pCVTZ optimized parameters, where “all-” denotes the correlation of all electrons.
iv. The effect of the inclusion of diffuse functions in the basis set is estimated as the difference between fc-MP2/aug-cc-pVTZ^24,51^ and fc-MP2/cc-pVTZ optimized parameters.

The jun-ChS variant^20^ is obtained by employing the partially augmented jun-cc-pV*n*Z basis sets^24,25^ (in the place of cc-pV*n*Z). Since these sets already incorporate diffuse functions, the iv term is not considered in conjunction with them. Instead, the iii term is the same in both approaches. If the contribution of diffuse functions (iv) is neglected in the original scheme, the “ChS-mAUG” model is obtained.

All DFT and MP2 calculations have been carried out using the Gaussian16 suite,^52^ while the CCSD(T) computations have been performed with the CFOUR package.^53^

## Results and Discussion

In agreement with the IUPAC definition of hydrogen bond,^54^ contrary to what used in ref (26), we denote as Imid–H_2_O the isomer where the imidazole ring acts as HBD, and as H_2_O–Imid the isomer where it acts as HBA.

### Computational Results

A preliminary scan of the potential energy surface (PES) of the molecular imidazole–water complex has been carried out at the B3 level and confirmed the DFT results reported in ref (26). Next, the geometries of Imid–H_2_O and H_2_O–Imid have been optimized at the rev-DSD, ChS, ChS-mAUG, and jun-ChS levels of theory. Their rev-DSD structures are sketched in Figure 1, while the geometrical parameters obtained at the different computational levels are collected in Table 1. From an inspection of the results collected in this table, we note that the four different approaches provide very similar results for the intramolecular parameters, with rev-DSD showing deviations within a few mÅ for distances and well within 0.1° for angles. Larger deviations, even within the different ChS variants, are noted for intermolecular parameters and, in particular, for the N···O distance. From Table 1, the cost-effectiveness of the rev-DSD level is evident: while providing results in nearly quantitative agreement with those issuing from composite schemes, its cost is comparable with the cheapest step of the latter approaches (i.e., MP2 in conjunction with a triple-ζ basis set).

**Table 1:** Equilibrium Geometries of H_2_O–Imid and Imid–H_2_O at Different Levels of Theory (Bonds in Å; Angles in deg)

H_2_O–Imid	rev-DSD	ChS	ChS-mAUG	jun-ChS	Imid–H_2_O	rev-DSD	ChS	ChS-mAUG	jun-ChS
C1–C2	1.3693	1.3652	1.3647	1.3647		1.3720	1.3679	1.3673	1.3673
N3–C1	1.3780	1.3755	1.3747	1.3749		1.3755	1.3731	1.3721	1.3723
C4–N3	1.3589	1.3553	1.3544	1.3547		1.3604	1.3569	1.3560	1.3562
N3–H5	1.0053	1.0031	1.0021	1.0022		1.0122	1.0086	1.0084	1.0085
C1–H6	1.0762	1.0741	1.0733	1.0735		1.0767	1.0747	1.0738	1.0740
C2–H7	1.0774	1.0753	1.0746	1.0747		1.0780	1.0758	1.0751	1.0753
C4–H8	1.0784	1.0769	1.0759	1.0761		1.0787	1.0764	1.0758	1.0760
C4–N9	1.3168	1.3122	1.3120	1.3122		1.3176	1.3132	1.3125	1.3125
O10–N9	2.8714	2.8470	2.8612	2.8594	O10–N3	2.9696	2.9773	2.9581	2.9644
O10–H11	0.9749	0.9733	0.9689	0.9684	O10–H12/13	0.9613	0.9582	0.9565	0.9565
O10–H12	0.9600	0.9569	0.9554	0.9554					
N3–C1–C2	105.26	105.28	105.26	105.27		105.44	105.45	105.46	105.46
C4–N3–C1	107.57	107.57	107.51	107.49		106.99	107.09	106.95	106.93
H5–N3–C4	126.10	126.06	126.12	126.12		126.39	126.39	126.43	126.41
H6–C1–C2	132.44	132.49	132.45	132.44		132.41	132.43	132.41	132.41
H7–C2–C1	128.20	128.28	128.17	128.17		128.03	128.15	128.03	128.02
H8–C4–C2	163.61	163.82	163.51	163.48		164.14	164.20	164.17	164.18
N9–C4–C2	38.17	38.16	38.23	38.25		38.53	38.46	38.57	38.59
O10–N9–C4	99.74	103.48	95.31	92.94	O10–N3–C1	126.92	126.72	126.87	127.42
H11–O10–C4	35.25	32.46	38.13	40.35	X11–O10–N3	163.51	187.85	172.45	174.07
H12–O10–H11	104.71	106.32	104.91	104.95	H12/13–O10–X11	52.64	52.95	52.74	52.71
O10–N9–C4–C2	174.76	171.56	174.38	176.24	H13–O10–H12	105.28	105.90	105.48	105.42
H11–O10–C4–C2	–4.86	–11.93	–4.59	–3.67	H12–O10–X11–C1	90.0	90.0	90.0	90.0
H12–O10–H11–C4	128.89	154.54	127.37	138.34	H13–O10–X11–C1	–90.0	–90.0	–90.0	–90.0

Unlike H_2_O–Imid, the structure of Imid–H_2_O is characterized by a symmetry plane containing the imidazole ring and the oxygen atom of the water molecule. Thus, Imid–H_2_O belongs to the *C*_*s*_ symmetry point group. In order to ease the interpretation of the geometrical parameters, we have used a dummy atom (X) placed on the HOH angle bisector to locate the water molecule with respect to the ring (see Table 1). In addition to the symmetry issue, the HB geometry presents some slight differences between the two isomers. On average, the computed N···O distance is about 0.11 Å longer in Imid–H_2_O than in H_2_O–Imid. Furthermore, the O–H bond in H_2_O–Imid and the N–H distance in Imid–H_2_O, which are involved in the HB donation, show stretches of about 13 and 6 mÅ, respectively, with respect to the isolated fragments. Concerning Imid–H_2_O, we note that, for the X11–O10–N3 angle, the original formulation of the ChS scheme leads to a value ∼15° larger than that obtained with jun-ChS and ChS-mAUG. This implies that ChS orients the water molecule differently. As expected, no significant differences are noted for the other geometrical parameters, the only exception being the HOH angle of water, which is overestimated at the ChS level in both H_2_O–Imid and Imid–H_2_O complexes. Noted is that the dihedral angles not explicitly reported in Table 1 are either 0° or 180°, as a consequence of the planarity of the imidazole ring.

For comparison purposes, we have also computed the structure of several complexes formed by water with N-containing heterocycles (Het) and reported the O···N distance at the rev-DSD level of theory (see Table 2). From the analysis of the results obtained, a systematic increase of the HB length when the heterocycle acts as HBD is noted, with a difference—by averaging on the two families—of 0.1 Å.

**Table 2:** Comparison between the O···N Distances in Several Het–H_2_O and H_2_O–Het Complexes at the rev-DSD Level (Bonds in Å)

heterocycle	*r*(O···N)
Het–H_2_O	
indole	2.99
pyrrole	3.00
imidazole	2.97
H_2_O–Het	
pyrazine	2.89
pyridazine	2.86
pyridine	2.89
pyrimidine	2.88
imidazole	2.87

Table 3 collects the rotational constants. While the *B*_e_’s have been straightforwardly derived from the equilibrium structures,^10^ the *B*_0_’s have been obtained by augmenting the former with the B3 vibrational corrections. Then, the *B*_0_’s can be directly compared with the experimental counterparts. For H_2_O–Imid, the ChS results are in remarkable agreement with the experiment,^26^ indeed showing an average relative error of 0.16%. The origin of the worse performance of both ChS-mAUG and jun-ChS models can be traced back to the slightly different water–imidazole relative position. In the H_2_O–Imid complex, the water molecule is characterized by a high mobility. As a consequence, different levels of theory lead to rather different values of the dihedral angles describing the position of water with respect to the imidazole ring. Deviations from the seemingly correct ChS value are noted when either neglecting diffuse functions (ChS-mAUG) or replacing the additive approximation by their systematic inclusion (jun-ChS), thus leading to larger errors on the *B* and *C* rotational constants. Moving to Imid–H_2_O, a remarkable agreement is found for all ChS variants, with the jun-ChS approach showing the best performance with an average relative error below 0.1%. Similarly to H_2_O–Imid, at different levels of theory, the N···O···X and O···N–C1 angles (which describe the water position) show differences when compared with each other, thus affecting the rotational constants determination. This is evident for the *A* constant of Imid–H_2_O: at the ChS level it deviates by 0.25%, while the relative discrepancy decreases by 1 order of magnitude moving to ChS-mAUG (0.02%) and jun-ChS (0.03%). This can be associated with the larger value, mentioned above, of *∠*(X11–O10–N3), in the case of ChS.

**Table 3:** Rotational Constants for H_2_O–Imid and Imid–H_2_O (All Parameters in MHz)

	rev-DSD	ChS	ChS-mAUG	jun-ChS	exp^26^
H_2_O–Imid					
*A*_e_	9481.5	9506.2	9519.3	9533.6	
*B*_e_	1830.2	1835.5	1866.8	1879.0	
*C*_e_	1539.9	1544.0	1567.0	1573.7	
*A*_0_	9439.7	9464.5	9477.5	9491.8	9502.79(43)
*B*_0_	1821.2	1826.6	1857.9	1870.1	1826.3929(13)
*C*_0_	1526.8	1531.0	1554.0	1560.7	1531.9303(10)
Imid–H_2_O					
*A*_e_	9504.5	9538.6	9564.3	9561.4	
*B*_e_	1673.9	1672.4	1684.5	1680.2	
*C*_e_	1432.7	1432.4	1441.8	1438.5	
*A*_0_	9462.8	9496.9	9522.6	9519.8	9520.99(26)^a^
*B*_0_	1657.8	1656.3	1668.4	1664.1	1662.91298(73)
*C*_0_	1417.2	1416.9	1426.3	1423.0	1420.74502(72)

a The 0^+^ state is reported.

Concerning rev-DSD, which is—as already mentioned—a level of theory affordable also for larger systems, the calculated *B*_0_^*i*^’s are in good agreement with the experimental ones, indeed showing a relative error of about 0.4% for both H_2_O–Imid and Imid–H_2_O complexes, which improves to 0.3% if one does not consider the *A* constant. In this respect, if we compare our rev-DSD/B3 results (with B3 referring to the vibrational corrections) with the current practice of directly using the *B*_e_^*i*^’s, evaluated from global-hybrid DFT calculations, for guiding spectral recording and analysis (as done, for example, in ref (26)), a reduction of the discrepancies ranging from a factor of 2 to more than 1 order of magnitude is evident.

Finally, the imidazole ring is characterized by the presence of two nitrogen atoms, which are quadrupolar nuclei. Having significantly different local environments, the corresponding quadrupole coupling interactions lead to distinctive spectroscopic features.^26^ Therefore, we decided to compare the experimental nuclear quadrupole coupling constants (χ_*ii*_’s) with the calculated ones. The results, reported in Table 4, show an overall good agreement with experiment at any level of theory considered, with the largest discrepancies being observed for the experimental data affected by large uncertainty. A remarkable improvement is noted when going from B3LYP^26^ to rev-DSD and CCSD(T)/jun-cc-pVTZ. Application of the jun-ChS approach only marginally changes the constants with respect to the latter level of theory.

**Table 4:** Nuclear Quadrupole Coupling Constants of H_2_O–Imid and Imid–H_2_O Computed at Different Levels of Thoery (All Parameters in MHz)

		theory^26^^,^^a^	rev-DSD	CC^b^	jun-ChS	jun-ChS+vib^c^	exp^26^
H_2_O–Imid							
N3	χ_*aa*_	1.192	1.102	1.136	1.173	1.120	1.143(19)
	(χ_*bb*_ – χ_*cc*_)	3.858	3.740	3.828	4.015	3.906	3.712(56)
N9	χ_*aa*_	–3.783	–3.099	–2.892	–2.880	–2.825	–2.889(21)
	(χ_*bb*_ – χ_*cc*_)	–0.490	–0.847	–0.895	–1.128	–1.147	–1.07(11)
Imid–H_2_O							
N3	χ_*aa*_	0.815	0.767	0.821	0.890	0.872	0.916(45)^d^
	(χ_*bb*_ – χ_*cc*_)	3.451	3.252	3.398	3.529	3.489	4.07(23)
N9	χ_*aa*_	–2.267	–2.052	–1.970	–2.069	–2.013	–1.859(44)
	(χ_*bb*_ – χ_*cc*_)	–2.680	–2.052	–2.354	–2.487	–2.475	–2.47(25)

a B3LYP-D3(BJ)/aug-cc-pVTZ level of theory.

b CCSD(T)/jun-cc-pVTZ level of theory.

c The jun-ChS equilibrium values have been augmented by the vibrational corrections at the B3 level.

d The 0^+^ state is reported.

### Semiexperimental Structure

As mentioned in the Computational Methodology section, the determination of the SE equilibrium structure for the two molecular complexes starts from a guess geometry obtained with the TMA. For the exploitation of the SE approach, the experimental rotational constants of four and three isotopic species (reported in ref (26)) for H_2_O–Imid and Imid–H_2_O, respectively, have been employed. The number of experimental data not being sufficient for a complete geometry evaluation, the LSF procedure has been applied to a reduced number of intermolecular structural parameters, while keeping the intramolecular and the other intermolecular parameters fixed at the guess values. Therefore, first of all, the accuracy of the equilibrium structures of the monomers, which are at the basis of the TMA, needs to be discussed.

Focusing on the isolated fragments (see Table 5), it is noted that all ChS schemes show a very good performance in terms of deviation from the SE equilibrium values. An inspection of Table 5 confirms the accuracy expected on the basis of the literature on this topic: 0.001–0.002 Å for bond lengths and 0.1–0.2° for angles.^44,45,55^ A slight overestimation of the water angle is observed at the ChS level. A good agreement is also found between the rev-DSD and SE values, with an average error of 3 mÅ and 0.15° for bond lengths and angles, respectively.

**Table 5:** Equilibrium Structures of Imidazole and Water (Bonds in Å; Angles in deg)

	SE*	rev-DSD	ChS	ChS-mAUG	jun-ChS
Imid					
C1–C2	1.3624	1.3704	1.3665	1.3658	1.3658
N3–C1	1.3741	1.3775	1.3750	1.3741	1.3743
C4–N3	1.3613	1.3629	1.3594	1.3586	1.3588
N3–H5	1.0016	1.0051	1.0029	1.0019	1.0020
C1–H6	1.0765	1.0765	1.0744	1.0735	1.0737
C2–H7	1.0755	1.0778	1.0755	1.0749	1.0750
C4–H8	1.0772	1.0785	1.0763	1.0756	1.0758
C4–N9	1.3101	1.3148	1.3106	1.3098	1.3098
N3–C1–C2	105.42	105.15	105.19	105.17	105.17
C4–N3–C1	107.01	107.30	107.34	107.25	107.22
H5–N3–C4	126.20	126.36	126.29	126.38	126.38
H6–C1–C2	132.65	132.57	132.60	132.58	132.59
H7–C2–C1	127.89	127.94	128.07	127.95	127.95
H8–C4–C2	164.36	164.34	164.34	164.34	164.35
N9–C4–C2	38.61	38.45	38.39	38.49	38.51
H_2_O					
O–H	0.9573	0.9610	0.9586	0.9563	0.9563
H–O–H	104.53	104.46	105.15	104.56	104.48

a Asterisk refers to the “Additional notes” section.

**Figure 2:**
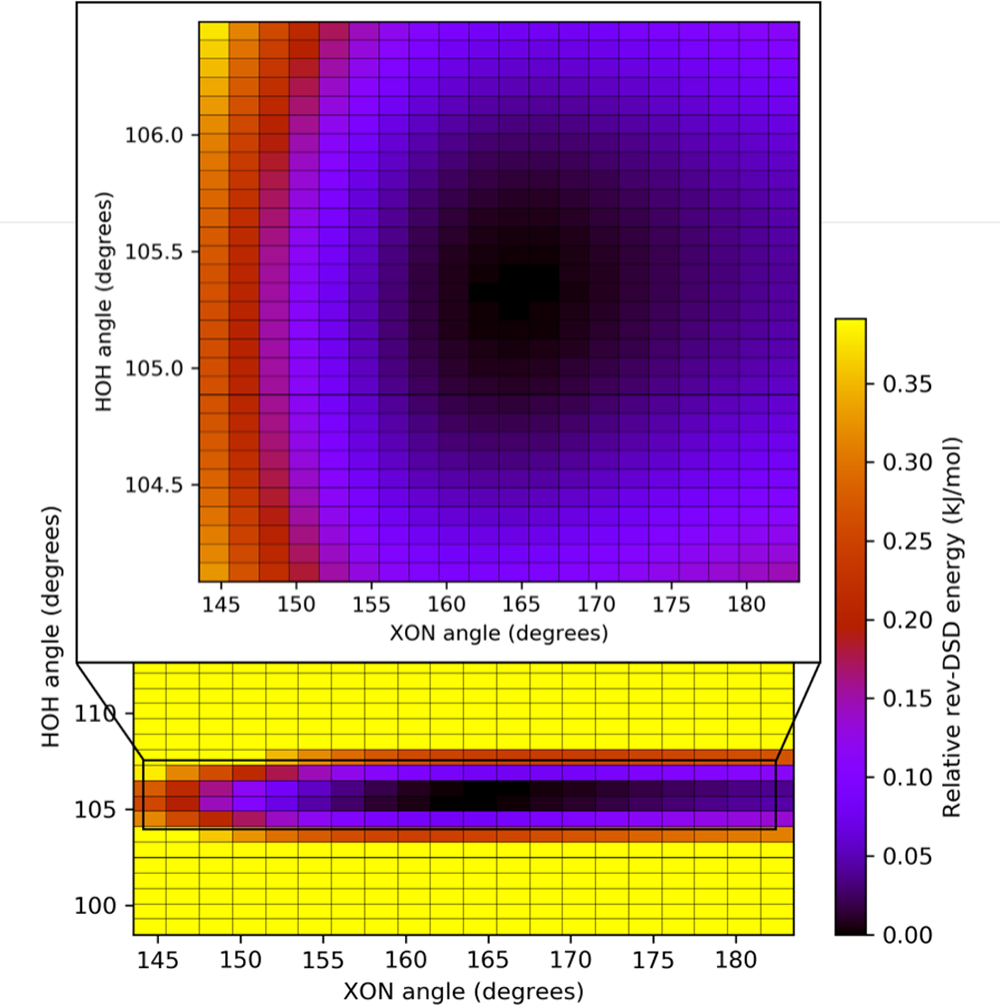
Two-dimensional scan of the PES near to the Imid–H_2_O minimum.

The LSF procedure has been carried out using the molecular structure refinement (MSR) software.^56^ For H_2_O–Imid, the fit has been carried out using all rotational constants equally weighted, while the *A*’s have been excluded from the fit of Imid–H_2_O. Different sets of determinable geometrical parameters have been tested in the LSF procedure in order to obtain the most robust fit. Among these tests, inclusion of *∠*(H–O–H) leads to precise (in terms of the statistical measures), but inaccurate, results for this angle. For instance, in the case of the Imid–H_2_O isomer, the use of the TMA-rev-DSD geometry in the LSF leads to *r*(N···O) = 2.96855(7) Å and *∠*(H–O–H) = 98.8(1)°, whereas the TMA-jun-ChS structure leads to *r*(N···O) = 2.96912(4) Å and *∠*(H–O–H) = 99.42(5)°. In order to better analyze these trends, a two-dimensional scan has been carried out at the rev-DSD level, thereby exploring the portion of the PES ruled by the *∠*(N···O···X) (143.5–183.5°) and *∠*(H–O–H) (from 98.5° to more than 115°) angles. As clearly evident in the bottom panel of Figure 2, those structures with *∠*(H–O–H) angles close to 105.5° are significantly more stable. To confirm the position of the minimum, a more focused second scan of the two-dimensional PES has been performed. The portion with *∠*(H–O–H) = 104°–106.5° has been probed, using the previous range for the other coordinate (see Figure 2). These investigations locate the minimum in the same position found in all geometry optimizations, therefore leading to the exclusion of the outcomes of this fitting procedure.

The source of the anomalous results addressed above may be traced back to the water mobility. Indeed, the presence of large amplitude motions has a huge impact on the geometry and questions the foundations of the SE approach, which relies on second-order vibrational perturbation theory. Experimental information on isotopic substitution at the hydrogen–water atoms would have helped in correctly deriving their positions within the molecular adduct. This cannot be accomplished by fitting instead intramolecular parameters such as the *∠*(H–O–H) angle, the overall conclusion being the exclusion of any structural parameter involving water hydrogens from our LSF procedure. A further evidence of this problem has been met in the fit of Imid–H_2_O. For this isomer, the value of the moment of inertia along the *a* axis (and, therefore, the rotational constant *A*) is strongly affected by the position of the hydrogen atoms of the water molecule, which, however, experience large amplitude motions. Within the VPT2 framework, the treatment of the latter represents a well-known issue,^57^ which has been confirmed, and partially solved, for this adduct by the exclusion of the *A*’s from the LSF procedure.

The results obtained by employing the TMA (fit 1) or theoretical (fit 2) intramolecular parameters are reported in Table 6 and confirm that the O···N distance is shorter in H_2_O–Imid than in Imid–H_2_O by at least 0.1 Å. The already mentioned difficulties in positioning the water molecule with respect to imidazole are at the origin of the slight differences in the SE equilibrium structure of H_2_O–Imid derived from the different fits. However, by comparing the results of fit 1 and fit 2 and taking into consideration the confidence interval of one standard deviation, we note that the fitted values are comparable. For Imid–H_2_O, the O···N distance as well as the *∠*(N···O···X) and *∠*(O···N–C1) angles are in remarkable agreement. Larger deviations are noted for the ChS results for both isomers, probably due to a greater difference in the “guess” values for the water molecule. Interestingly, the comparison of the results of fit 1 points out the strength of the TMA, which is able to provide reliable geometries even when the reference structure is obtained at the DFT level (instead of a computational expensive CC-based composite scheme).

**Table 6:** Semiexperimental Equilibrium H_2_O–Imid and Imid–H_2_O Intermolecular Parameters (Bonds in Å; Angles in deg)^a^

	method	guess value	fit 1	fit 2^b^
H_2_O–Imid				
*r*(O···N)	rev-DSD	2.8714	2.86(2)	2.771(8)
	ChS	2.8470	2.80(2)	2.79(1)
	ChS-mAUG	2.8612	2.85(2)	2.87(2)
	jun-ChS	2.8594	2.82(4)	2.83(4)
*∠*(O···N–C4)	rev-DSD	99.74	101(3)	121(4)
	ChS	103.48	112(5)	117(5)
	ChS-mAUG	95.31	102(3)	100(3)
	jun-ChS	92.94	106(7)	104(7)
Imid–H_2_O				
*r*(N···O)	rev-DSD	2.9696	2.987(2)	2.9637(4)
	ChS	2.9773	2.981(1)	2.981(2)
	ChS-mAUG	2.9581	2.987(2)	2.995(2)
	jun-ChS	2.9644	2.986(2)	2.992(2)
*∠*(O···N–C1)	rev-DSD	126.92	137.0(6)	125(1)
	ChS	126.72	134.6(6)	133.6(7)
	ChS-mAUG	126.87	137.1(7)	138.4(7)
	jun-ChS	127.42	136.8(7)	137.9(7)
*∠*(N···O···X)	rev-DSD	163.51	155.3(6)	157.7(7)
	ChS	187.85	161.4(6)	162.0(7)
	ChS-mAUG	172.45	155.5(6)	155.4(7)
	jun-ChS	174.07	155.6(6)	155.4(6)

a The error within brackets is one standard deviation.

b No TMA has been used to correct the intramolecular parameters.

### Energetics

The energetic characterization of the two isomers has been carried out at both the jun-ChS and rev-DSD levels, with the former model for interaction energies being fully described in ref (20). Actually, the different “cheap” expressions can be derived from eq 4 by replacing the geometrical parameter *r* with the total energy. In particular, the jun-ChS model chemistry is proven to provide a very good compromise between accuracy and computational cost in the description of noncovalent interactions.^20^ Furthermore, two different reference geometries (rev-DSD and jun-ChS) have been used in order to determine the influence of the structure on the energetics. The results are reported in Table 7. According to them, H_2_O–Imid is about 6.2 kJ/mol more stable than the Imid–H_2_O isomer, in agreement with both a previous FTIR experiment^27^ and the findings of ref (26). With respect to the relative stability, the effect of the different geometry on the energy is negligible (i.e., less than 0.1 kJ/mol).

**Table 7:** Energetics of the Two Imidazole–Water Complexes (All Results in kJ/mol)

			int en (jun-ChS)		int en (rev-DSD)	
	ref geom	rel en (jun-ChS)	CP	NCP	CP	NCP
H_2_O–Imid	rev-DSD	0.00	–32.54	–32.76	–31.34	–32.91
	jun-ChS	0.00	–32.24	–32.45	–30.83	–32.38
Imid–H_2_O	rev-DSD	6.20	–25.89	–25.97	–24.61	–25.97
	jun-ChS	6.27	–25.77	–25.86	–24.45	–25.80

The jun-ChS and rev-DSD levels have also been employed to evaluate the interaction energy (int en), possibly accounting for the basis set superposition error (BSSE) by means of the counterpoise (CP) correction.^58^ The results (Table 7) show a good agreement between the two levels of theory (within 0.1 kJ/mol) when CP corrections are not incorporated (denoted as NCP). As expected, NCP and CP results are quite similar to one another at the jun-ChS level (within 0.2 kJ/mol) because of the extrapolation to the CBS limit, whereas the CP correction significantly worsens (by about 1.5 kJ/mol) the rev-DSD results (with respect to jun-ChS). It is thus confirmed that jun-ChS computations (for both energies and geometries) can be safely performed without any CP correction. The same applies to rev-DSD results, possibly due to to a fortuitous error compensation and/or the conditions employed in the parametrization of the functional.

From a close inspection of the results of Table 7, a conclusion on the deformation experienced by the monomers (from isolated to part of the adduct) can be drawn. The difference between the jun-ChS-CP interaction energies of the two isomers (6.65 and 6.46 kJ/mol at the rev-DSD and jun-ChS geometries, respectively) is mainly ascribable to the relative jun-ChS electronic energy (6.20 and 6.27 kJ/mol at the two reference geometries). The remaining contribution (0.45 and 0.20 kJ/mol), due to the deformation of the monomers, slightly favors the Imid–H_2_O isomer.

While the interaction energy clearly points out a stronger interaction in H_2_O–Imid, a deeper insight on the origin of different HB patterns can be gained by the natural energy decomposition analysis (NEDA).^59^ This has been carried out at the B3 level using the NBO 7.0^60^ suite of programs interfaced to the latest revision of the Gaussian16 program. Furthermore, in order to obtain general information, such an analysis has been extended to different N-heterocycle–water adducts. The results, collected in Table 8, show a close agreement between B3 (last column) and rev-DSD (last column of Table 7) total energies for the case of imidazole and point out a clear trend of the two hydrogen bond patterns (Het–H_2_O and H_2_O–Het). When the N-heterocycle molecule acts as HBD, the interaction is weaker than that experienced in those adducts where water acts as HBD, this outcome being in agreement with the basicity of the heterocycles and the O···N distances reported in Table 2. Within the two groups, no significant difference in the various contributions to the total interaction energy can be noted. Focusing on the total energy, the results within the two group are in line with the acidity and basicity of imidazole, which always shows the largest interaction energy. Indeed, the p*K*_A_ value of imidazole is 14.5, thus being more acidic than pyrrole. On the other hand, the p*K*_A_ value of the conjugated acid is ∼7, which means that imidazole is about 60 times more basic than pyridine.

**Table 8:** Energy Decomposition Analysis^a^ for Het–H_2_O and H_2_O–Het Complexes (Values in kJ/mol)

heterocycle	El^b^	CT^c^	core	total
Het–H_2_O				
indole	–45.0	–44.2	65.8	–23.4
pyrrole	–42.1	–40.4	60.7	–21.8
imidazole	–47.2	–44.7	66.7	–25.2
H_2_O–Het				
pyrazine	–55.8	–49.4	78.2	–27.0
pyridazine	–61.7	–57.2	87.5	–31.4
pyridine	–60.8	–60.0	89.3	–31.5
pyrimidine	–58.2	–50.8	80.6	–28.4
imidazole	–63.7	–58.9	89.2	–33.4

a Calculated at the B3LYP-D3(BJ)/jun-cc-pVTZ level of theory, using rev-DSD optimized geometries.

b Electrostatic plus polarization interaction.

c Charge transfer.

The two attractive terms, the electrical interaction (which includes the electrostatic and polarization contributions) and the charge transfer, are similar, with the maximum discrepancy being smaller than 7.5 kJ/mol. Furthermore, similar values are also found for the repulsive core interaction, with average values of 64.4 and 85.0 kJ/mol for Het–H_2_O and H_2_O–Het, respectively. In both the investigated groups, imidazole is found to have the strongest interaction with the water molecule.

## Conclusions

A computational characterization of H_2_O–Imid and Imid–H_2_O, two isomers of the imidazole–water complex, has been carried out using different levels of theory for the geometry optimization, also aiming to test the reliability of the jun-ChS composite scheme and the DSD-PBEP86 double-hybrid density functional in its most recent reparametrization. To check the performance of these two levels of theory, we relied on the results obtained in a recent experimental work based on rotational spectroscopy.^26^ Indeed, rotational constants intrinsically contain structural information. We proceeded in two different ways: we derived the rotational constants from our structures to be compared with the experimental ones and we used the latter, available for different isotopic species, in the semiexperimental approach for deriving accurate structures (to be compared with the computed ones). Both strategies required the computation of the vibrational corrections to rotational constants (either to be added to the computed equilibrium rotational constants or to be subtracted from the experimental ground-state rotational constants), which have been determined from B3LYP-D3(BJ)/jun-cc-pVDZ anharmonic calculations within the VPT2 model. While both strategies pointed out the accuracy of the jun-ChS model applied to geometries and the reliability of the rev-DSD level of theory, some discrepancies have been found that could be ascribed to the high flexibility of the water molecule position within the adduct. Further developments in the treatment of large amplitude motions will be addressed in a future work.

The semiexperimental equilibrium structure of the two isomers has been determined by means of a least-squares fit of the SE equilibrium moments of inertia for several isotopologues, with the intramolecular parameters being kept fixed. The values used for the latter have been derived from the application of the template model approach. One important finding is that the use of this approach leads to robust and reliable results, which are independent of the level of theory employed. The overall conclusion is that the rev-DSD-PBEP86-D3(BJ)/jun-cc-pVTZ level of theory in conjunction with the TMA is a valuable choice for the determination of the equilibrium structure of medium-sized molecular complexes at a reasonable computational cost.

Finally, an accurate energetic characterization of the imidazole–water complexes has been carried out. In addition, their energy decomposition analysis (EDA) is reported also for several N-heterocycle complexes with water for comparison purposes. The EDA pointed out that the different contributions are essentially unchanged within the series of adducts considered.

In our opinion, together with the interest of the studied system, the proposed computational strategy paves the route toward accurate structural and energetic characterizations for large noncovalent complexes of current technological and biological interest.

## Footnotes

a The SE equilibrium structures of imidazole and water are available for download from smart.sns.it.
